# Supplementary material for: A RAD51–ADP double filament structure unveils the mechanism of filament dynamics in homologous recombination
Source: Nat Commun. 2023 Aug 17;14:4993. doi: 10.1038/s41467-023-40672-5 (PMC10435448; doi:10.1038/s41467-023-40672-5)
Supplement: Supplementary file 1 — Supplementary Information [file 41467_2023_40672_MOESM1_ESM.pdf]

Supplementary Information

Supplementary Table 1. Cryo-EM data collection, refinement and validation statistics

	RAD51-ADP<br>(EMDB-34372 and EMD-36040)<br>(PDB 8GKY)
**Data collection and processing**	
Magnification	165,000
Voltage (kV)	300
Electron exposure (e–/Å^2)	50
Defocus range (μm)	-1.0 to-2.0
Pixel size (Å)	0.83
Symmetry imposed	Helical
Total particle images (no.)	2,622,222
Box size (pixel)	384
Rise (Å)	9.8
Twist	-155.6^o
Map resolution (Å)	3.14
FSC threshold	0.143
Map resolution range (Å)	3.14-6.6
**Refinement**	
Initial model used (PDB code)	5H1B
Model resolution (Masked, Å)	3.33
FSC threshold	0.5
Map sharpening *B* factor (Å^2)	-152.1
Model composition	
Non-hydrogen atoms	18,4467
Protein residues	2375
Nucleotide	0
Ligands: ADP, Mg	8, 8
B factors (Å^2)	
Protein	82.03
Nucleic acid	-
Ligand	88.88
R.m.s. deviations	
Bond lengths (Å)	0.003
Bond angles (°)	0.757
Validation	
MolProbity score	2.11
Clash score	12.75
Poor rotamers (%)	0
Ramachandran plot	
Favored (%)	91.88
Allowed (%)	7.31
Disallowed (%)	0.81

Supplementary figure 1. Comparison of hRAD51–ATP and hRAD51–ADP filaments. Representative micrographs and 2D class average images and electron density maps of hRAD51–ATP and hRAD51–ADP filaments are shown on the left and right, respectively. The corresponding diameters and helical pitches are indicated.

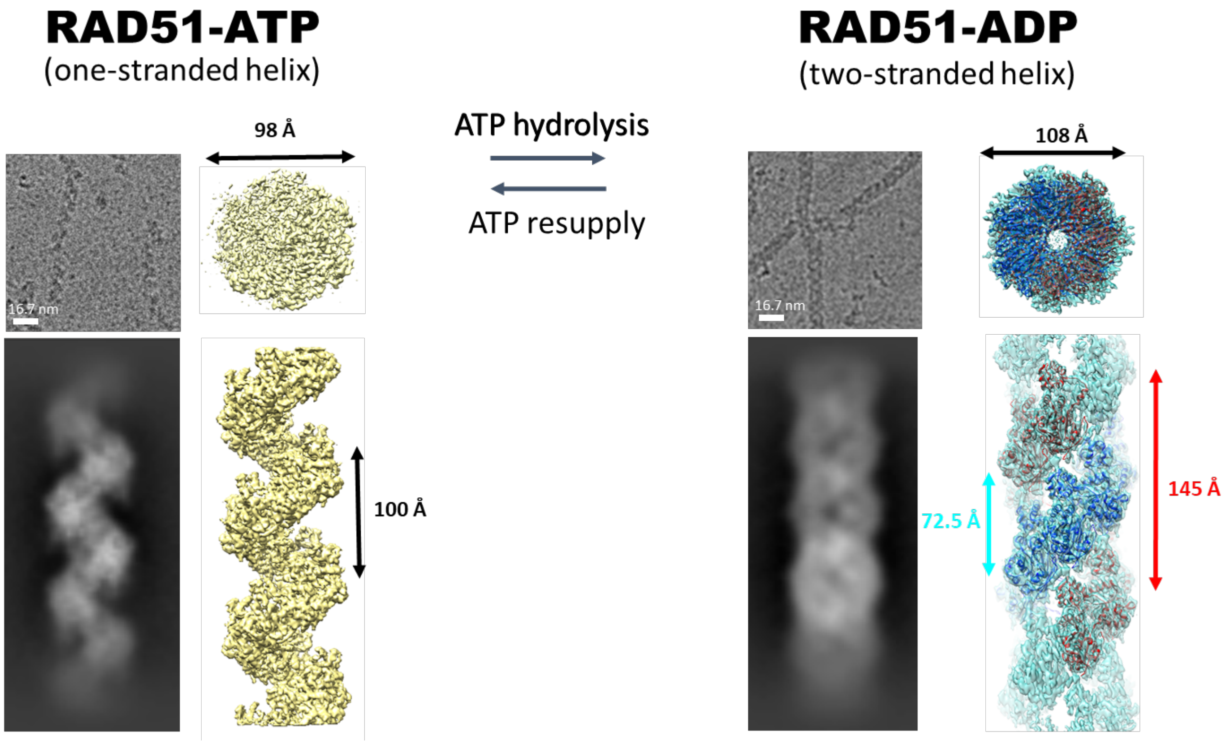

Supplementary figure 2. Flow chart for the helical reconstruction of ssDNA bound hRAD51–ADP double-filament. Workflow for structure determination. A representative cryo-EM micrograph and a representative 2D class average are shown in the top panel. A representative box size for particle picking and 2D class average is shown as a white box. Initial helical parameters (rise 19.6 Å and twist 46.7º) of one-stranded filament with a helical pitch of 145 Å yielded only a 3.69 Å resolution map. By applying a two-stranded filament with a helical pitch of 72.5 Å and focused refinement, the map resolution was improved to 3.14 Å. Representative cryo-EM maps superimposed with the refined structures are shown in the middlebox. The density map of the focused refinement colored by local resolution and the composite map are shown in the bottom left. The final average map resolution for the overall density of the filament is shown as calculated in cryoSPARC by the gold standard technique. FSC curves of the refined model versus summed map (black) and the refined model versus the half map (red) were calculated by Phenix.

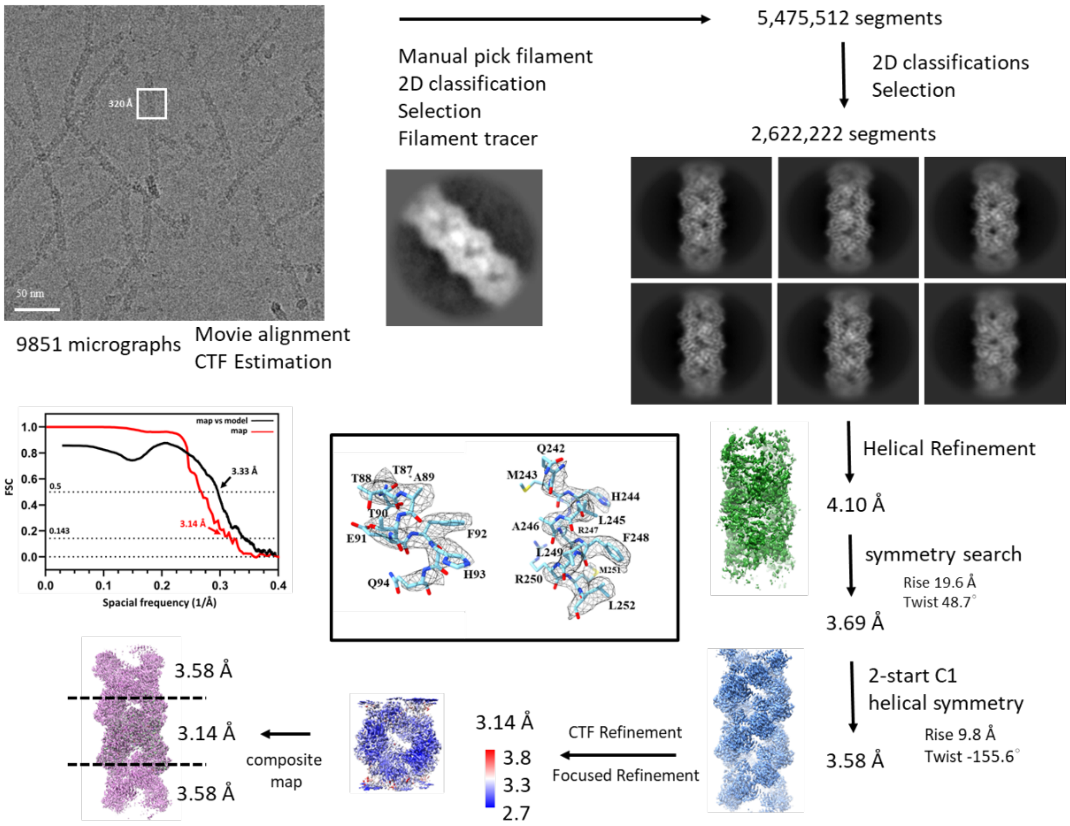

Supplementary figure 3. Flow chart for the helical reconstruction of dsDNA bound hRAD51–ADP double-filament. Workflow for structure determination. A representative cryo-EM micrograph and representative 2D class average images are shown in the top panel. The helical refinement in cryoSPARC without inputs of helical paraments yields a cryo-EM map with 18Å resolution estimated by the d99 program in Phenix. The top and side (bottom right and center) view docked with hRAD51–ADP filament model generated from this work indicates that the dsDNA bound hRAD51–ADP filament is a double-filament. The slice view (bottom left) shows the dsDNA density (colored in yellow) inside the hRAD51 filament. The filament was assembled in a buffer (35 mM Tris-HCl pH 7.5, 108 mM KCl, and 1 mM DTT) containing 1 mM ADP, 0.05 mM ATP, 2.5 mM MgCl2 at 37℃ for 30 min.

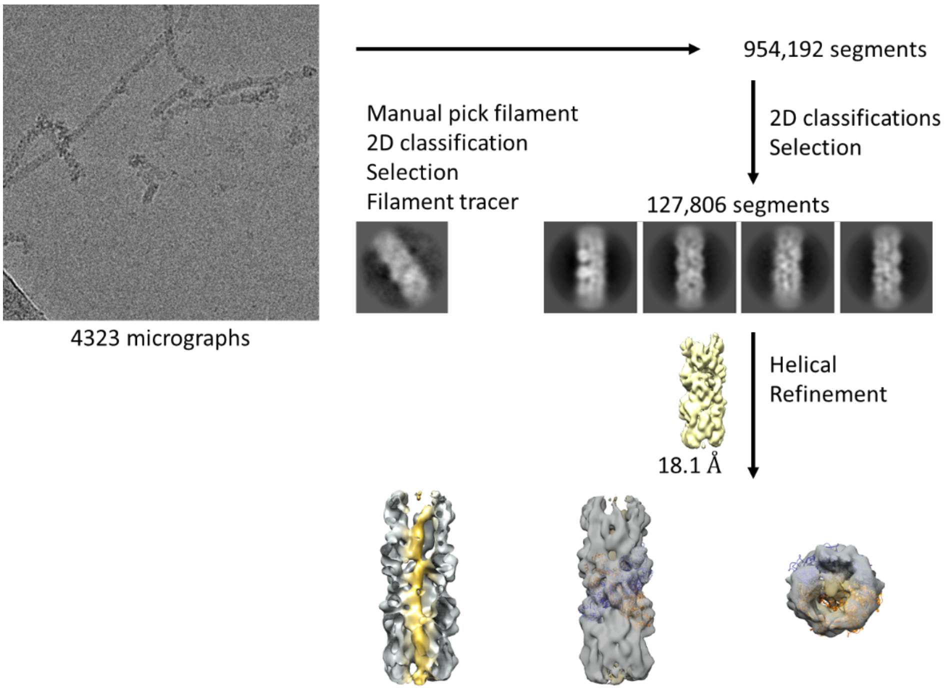

Supplementary Figure 4. Overlay of hRAD51–ATP (grey) and hRAD51–ADP (cyan) protomers

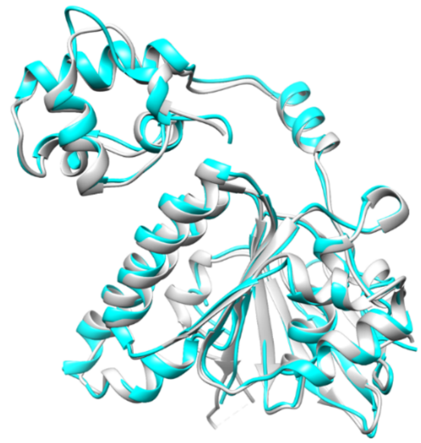

Supplementary figure 5. Positively-charged surface potentials inside both hRAD51–ATP and hRAD51–ADP filaments. A side view (left) and central slice (right) of the surface potentials of (A) hRAD51–ATP and (B) hRAD51–ADP filaments. Negative-, neutral-, and positive- charged residues are colored red, white, and blue, respectively. The side view shows that the hRAD51–ADP filament is more tightly packed compared to the hRAD51–ATP filament. The surface potentials are calculated by UCSF ChimeraX.

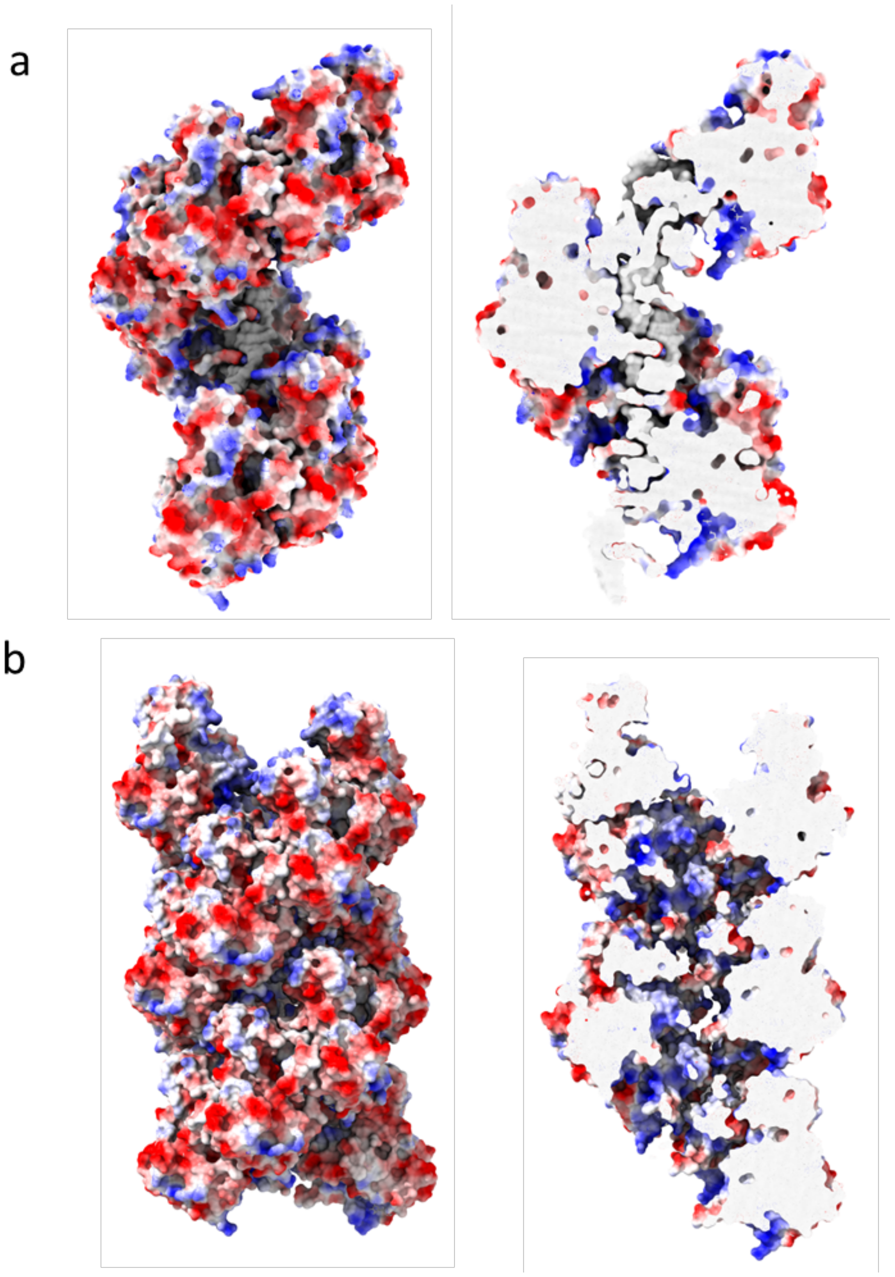

Supplementary figure 6. ssDNA inside the hRAD51 double-filament. The cryo-EM map of ssDNA bound hRAD51–ADP double-filament at the contour level of 0.22 (a) and 0.12 (b). The one filament of hRAD51 double-filament is colored in cyan and the other is colored in brown. The slice view is shown on the right to show the density inside the double-filament. At the low contour level, ssDNA density (colored in blue) can be seen inside the hRAD51-double filament.

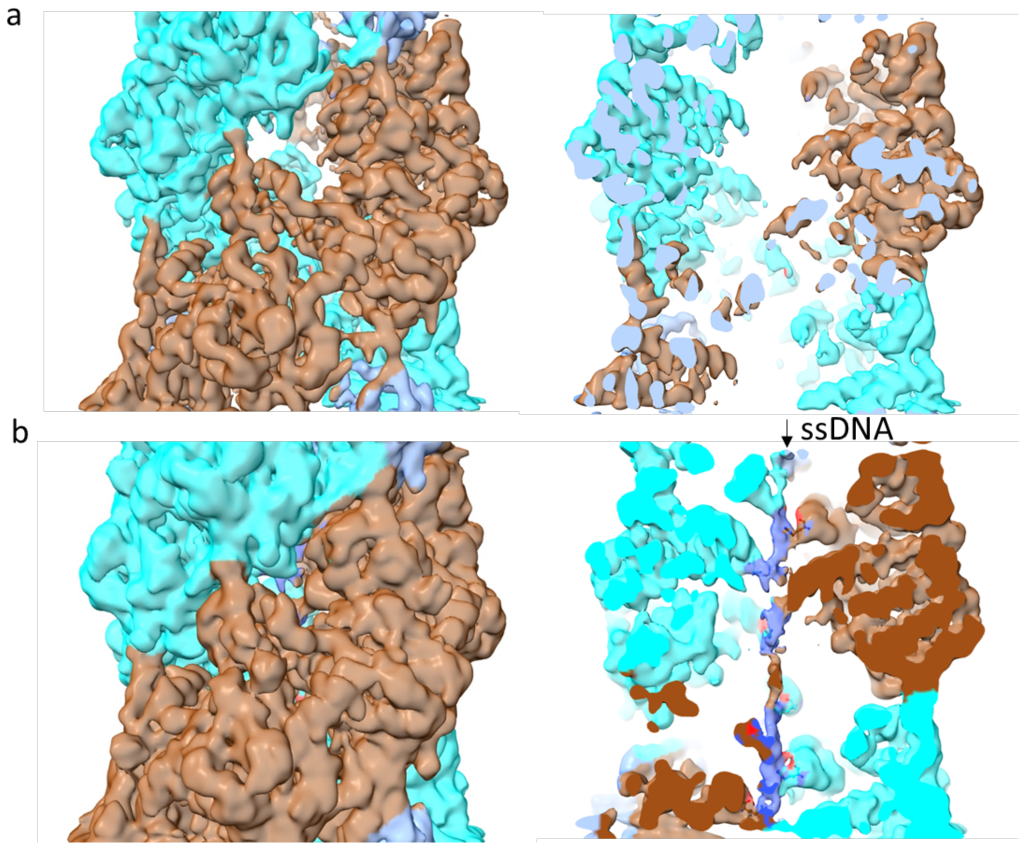

Supplementary figure 7. The presence of ssDNA is required to form hRAD51–ADP double-filaments. (a) Two representatives of cryo-EM micrograms of ADP bound hRAD51 in the absence of ssDNA. No hRAD51–ADP double-filament can be observed. Only ring-shaped or protein aggregated, indicated by red and white arrows, respectively, can be observed. (b) Negative stain TEM visualization of ssDNA bound hRAD51–ADP double-filaments. 80-nt DNA is conjugated with a 10 nm nanogold particle shown as a black dot. 10 nm nanogold particles aside with RAD51–ADP double-filaments can be observed, indicating that hRAD51 forms nucleoprotein filament with ssDNA. 50 nm scale bars are indicated in each microgram.

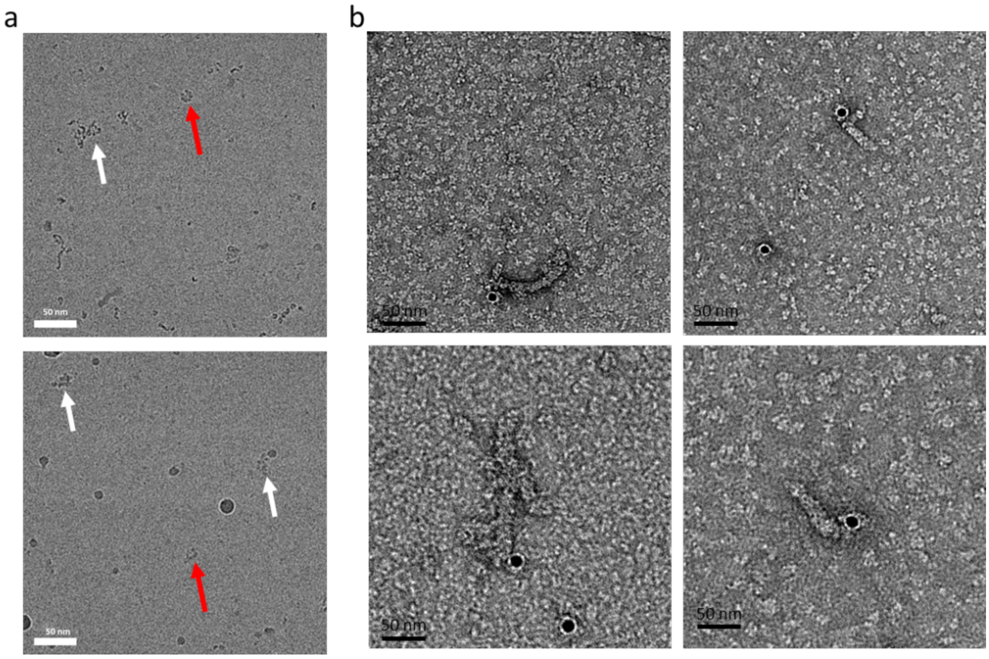

Supplementary figure 8. hRAD51–ADP double-filament interface. One protomer from one RAD51–ADP single-filament and one protomer from the other filament are shown as cyan and red ribbon diagrams, respectively. The side chains of R130, R303, K313, and R130* (from the adjacent protomer) are shown as blue sphere diagrams. The region of site II and the N-terminal domain (NTD) are labeled.

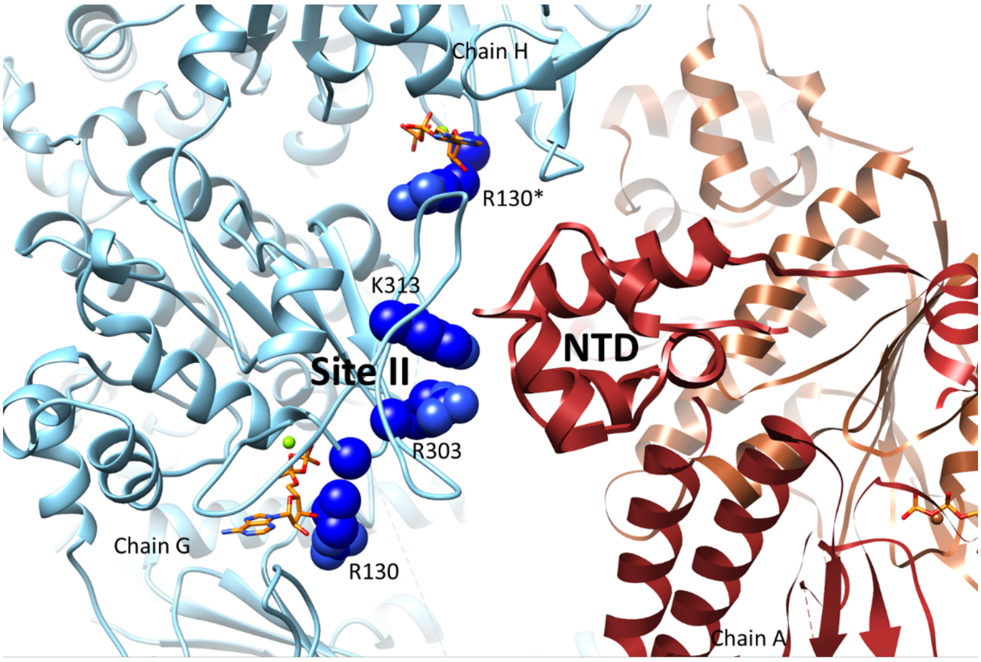

Supplementary figure 9. Local resolution of hRAD51 protomer. The cryo-EM of one representative hRAD51 protomer is colored by local resolution. NTD and site II regions are highlighted in black dashed line boxes. The map quality of NTD and site II regions located at the double-filament interface are the poorest.

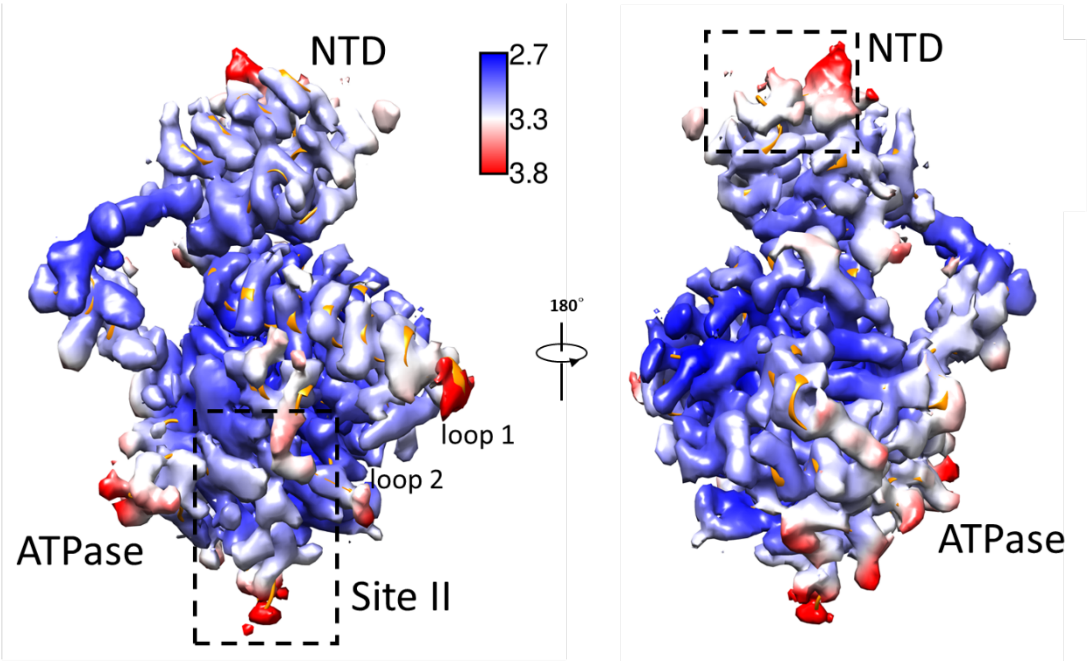

Supplementary figure 10. Overlay of hRAD51–ADP with hRAD51–ATP filaments. Four protomers from the hRAD51–ADP filament are shown as gray ribbon diagrams and are labeled with numbers 1 to 4. The corresponding four protomers from the hRAD51–ATP filament and additional four protomers are colored gray with the first protomer of the hRAD51–ATP filament overlaid with the first protomer of the hRAD51–ADP filament. The bound ssDNA is shown as a blue ribbon diagram. The overlaid structures show that the fourth protomer from hRAD51–ADP completely detaches from the fifth promoter of hRAD51–ATP filament.

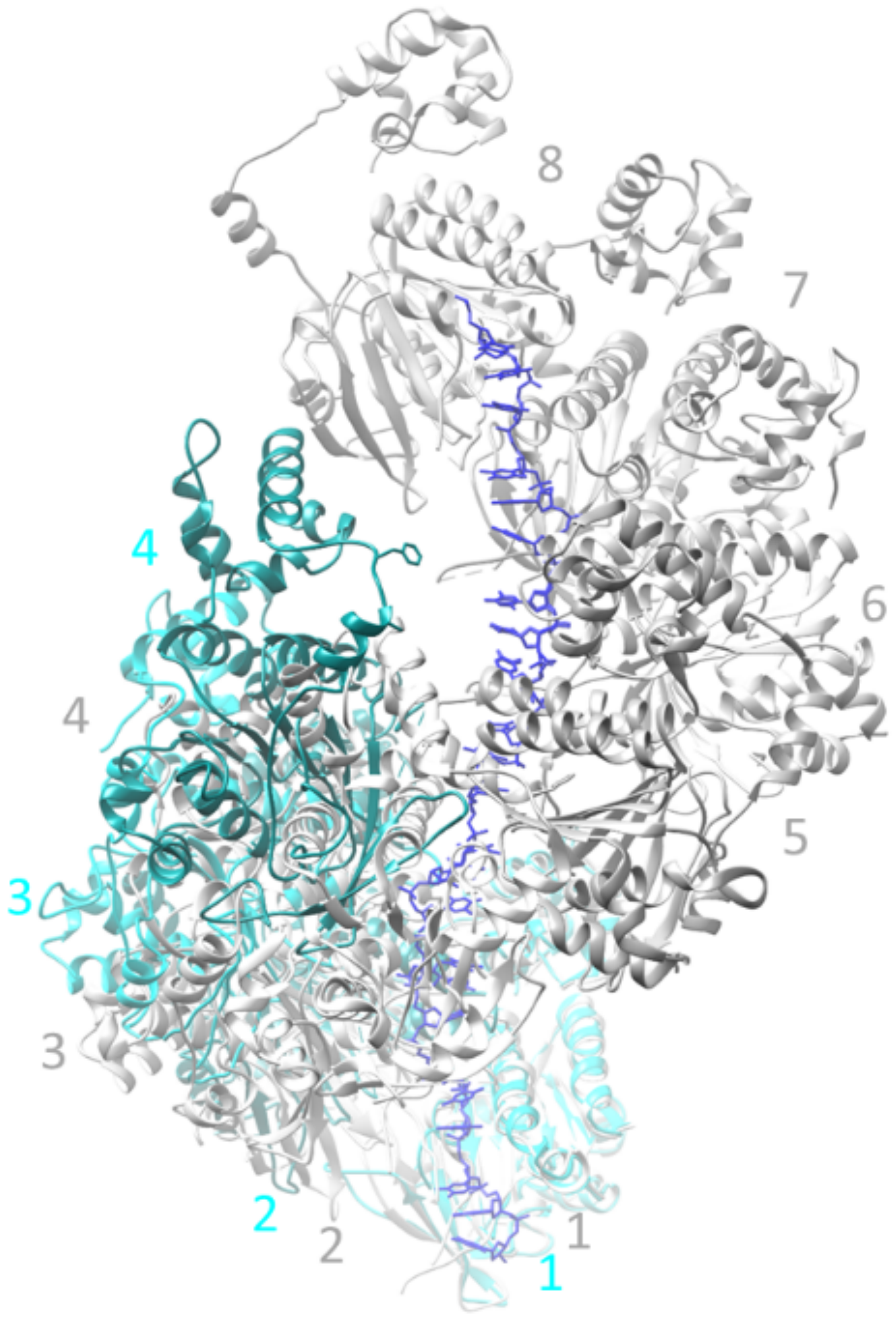

Supplementary figure 11. 2D class averaging of ADP/ATP-bound intermediates. Representative 2D class averaging from cryoSPARC (a) and RE2D (b) is shown. Simulated 2D projections from the ADP/ATP-bound intermediates are shown in (c) and the 2D class images shown in B that resemble those projections are highlighted in the green and orange boxes.

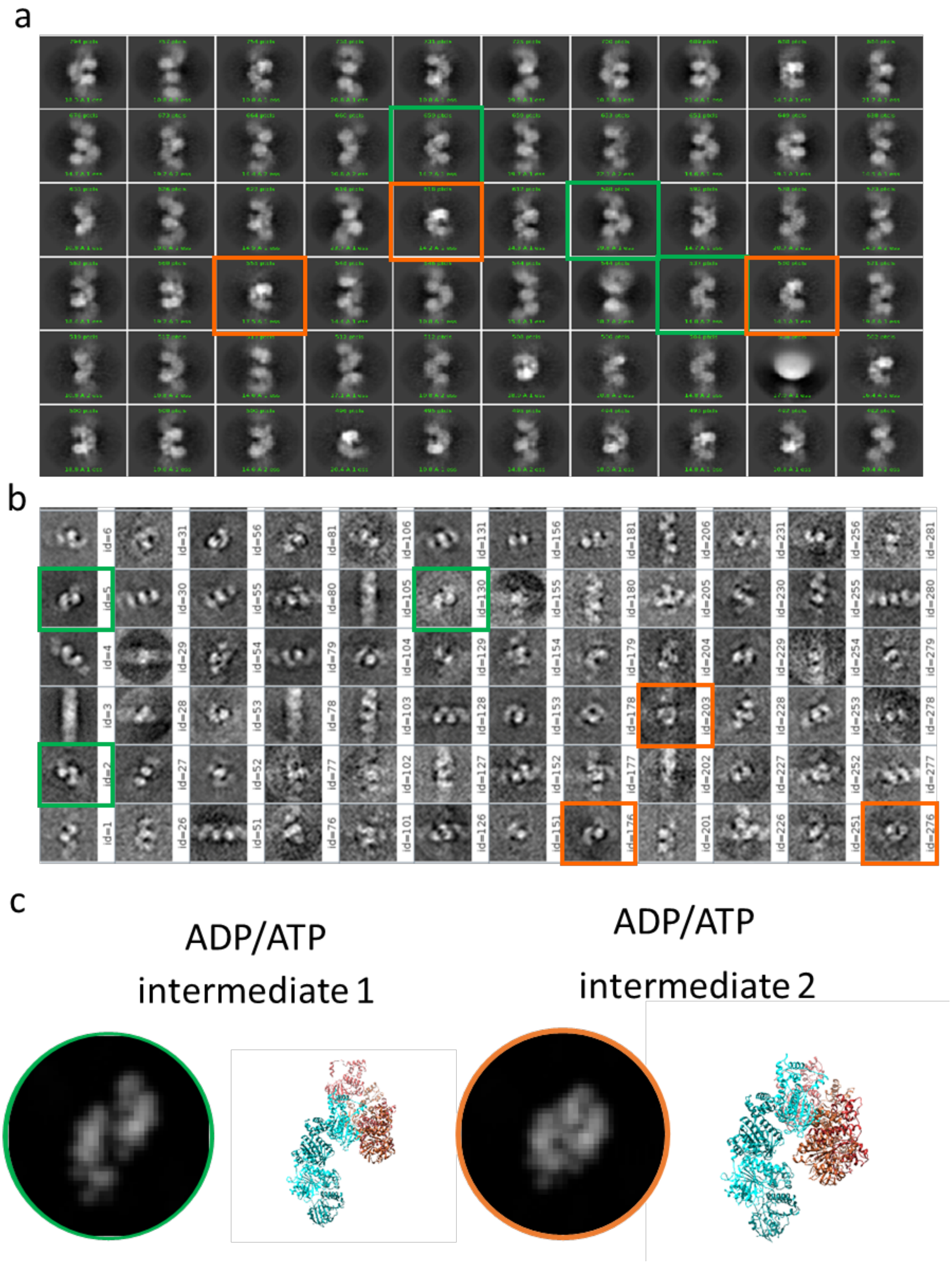
a
b
c
ADP/ATP
intermediate 1
ADP/ATP
intermediate 2

Supplementary figure 12. Flowchart of single particle reconstruction of intermediate 1 and 2 shown in Figure 4. Data process of reconstruction of intermediate 1 and intermediate 2 by cryoSPARC. A presentative microgram with a zoom-in view of particle boxes are shown. Low-resolution cryo-EM maps superimposed with the proposed intermediate models are shown in the bottom right. The resolution of both cryo-EM maps is estimated to be ~ 18Å based on the d99 software in the Phenix program. Both EM map and coordinate can be downloaded from https://doi.org/10.6084/m9.figshare.20920903

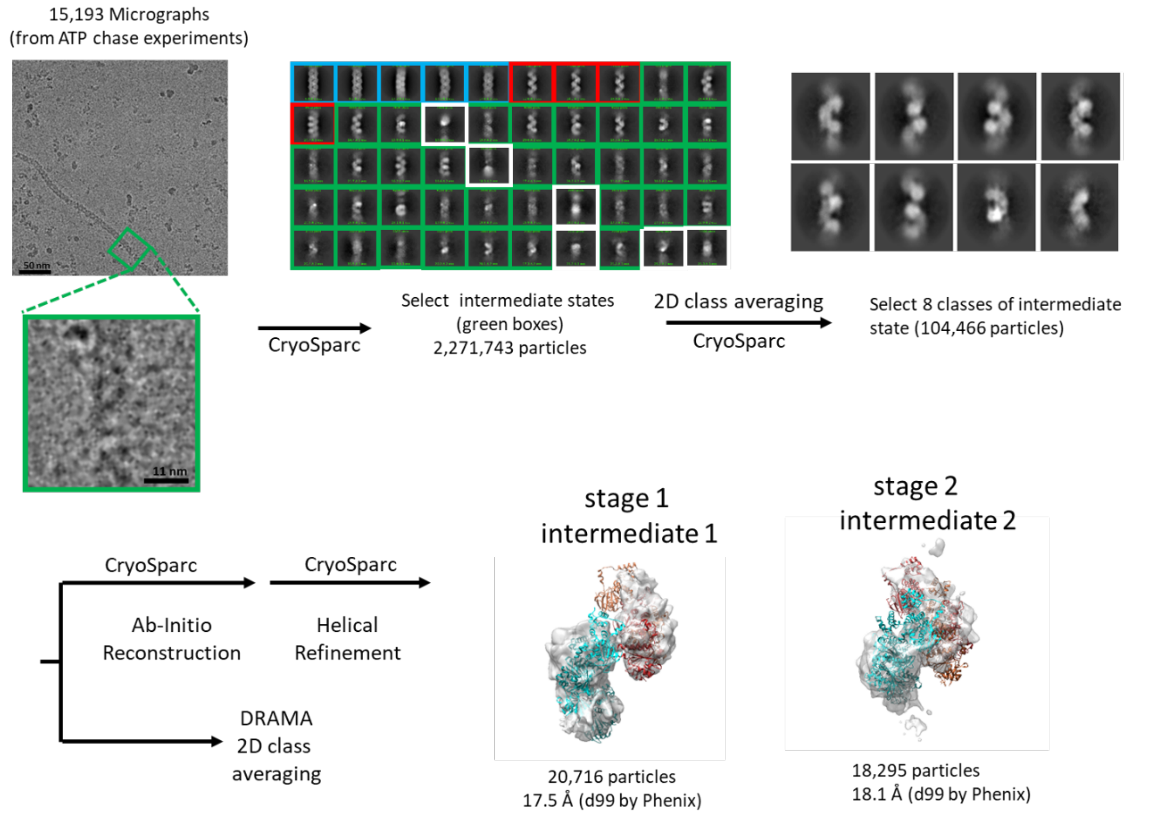

Supplementary figure 13. Stimulated intermediate structures in collapsing mechanism and the corresponding cryo-EM work. Simulated structures with representative side views and 2D projections from intermediate 1 and intermediate 2 in Figure 4 are shown on the left. Corresponding 2D projections from class averaging analysis are shown on the right. The simulated structures are shown docked into low-resolution molecular envelopes reconstructed by cryo-EM analysis.

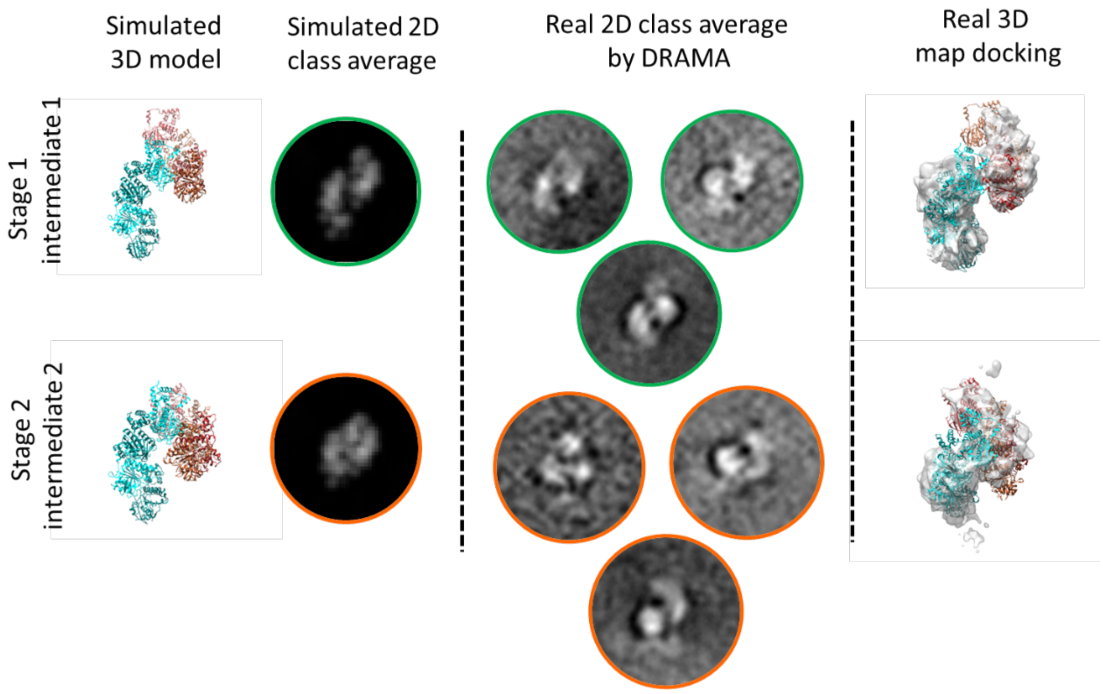

Supplementary figure 14. 2D class average images resembling 2D projections of the intermediate steps

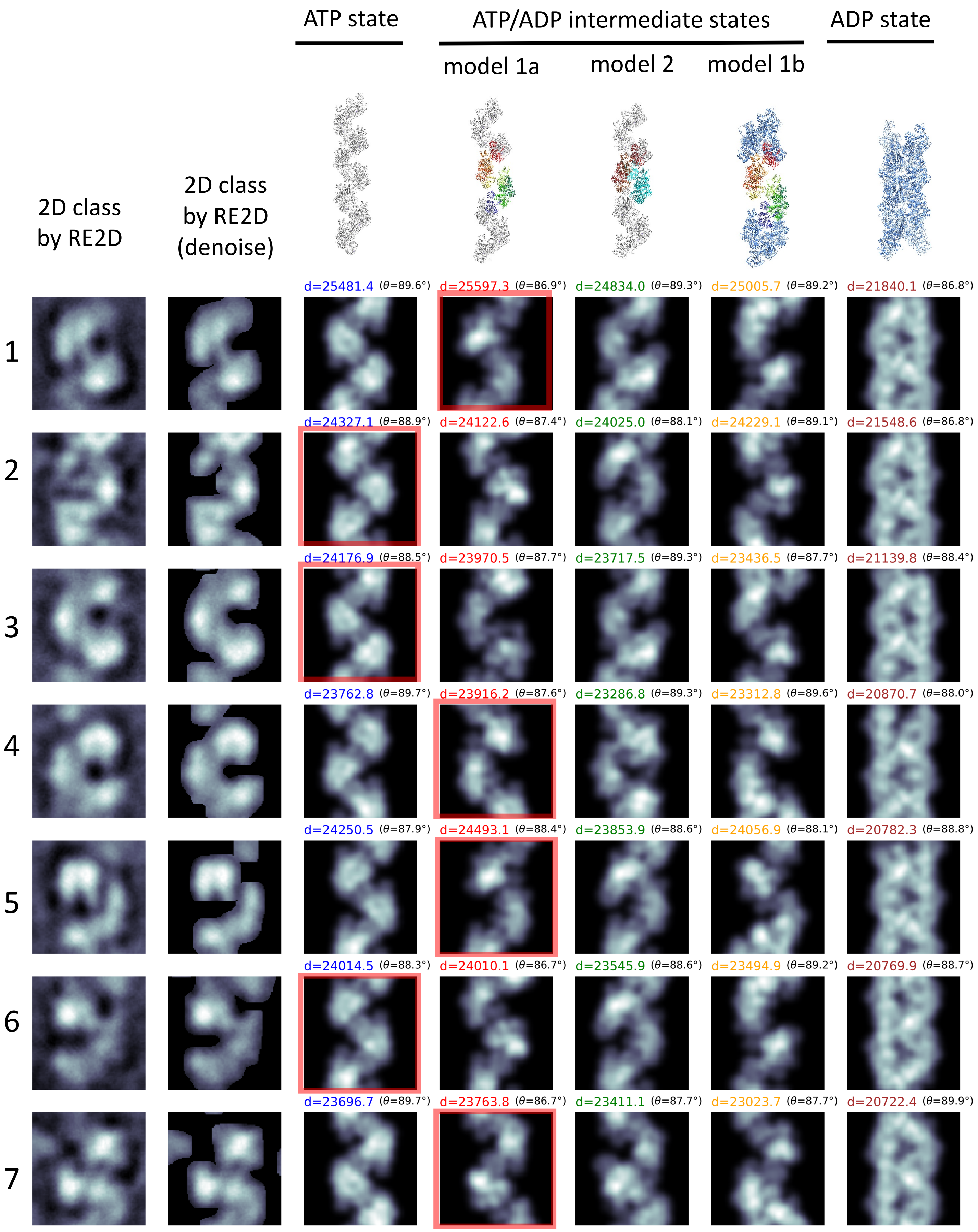

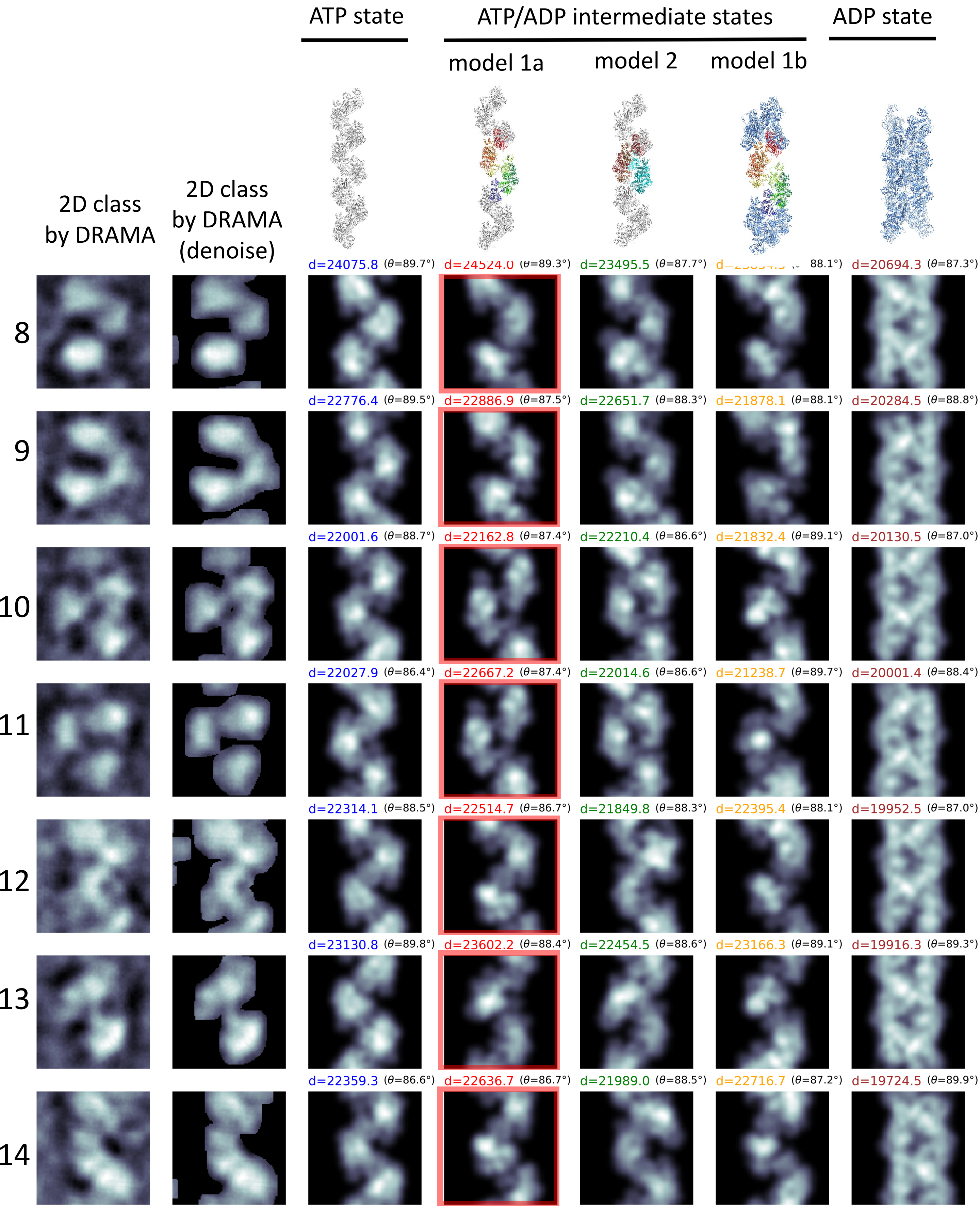

ATP state
ATP/ADP intermediate states
ADP state
model 1a
model 2
model 1b
2D class by DRAMA
2D class by DRAMA (denoise)
d=24075.8 (θ=89.7°)
d=24524.0 (θ=89.3°)
d=23495.5 (θ=87.7°)
88.1°)
d=20694.3 (θ=87.3°)
8
d=22776.4 (θ=89.5°)
d=22886.9 (θ=87.5°)
d=22651.7 (θ=88.3°)
d=21878.1 (θ=88.1°)
d=20284.5 (θ=88.8°)
9
d=22001.6 (θ=88.7°)
d=22162.8 (θ=87.4°)
d=22210.4 (θ=86.6°)
d=21832.4 (θ=89.1°)
d=20130.5 (θ=87.0°)
10
d=22027.9 (θ=86.4°)
d=22667.2 (θ=87.4°)
d=22014.6 (θ=86.6°)
d=21238.7 (θ=89.7°)
d=20001.4 (θ=88.4°)
11
d=22314.1 (θ=88.5°)
d=22514.7 (θ=86.7°)
d=21849.8 (θ=88.3°)
d=22395.4 (θ=88.1°)
d=19952.5 (θ=87.0°)
12
d=23130.8 (θ=89.8°)
d=23602.2 (θ=88.4°)
d=22454.5 (θ=88.6°)
d=23166.3 (θ=89.1°)
d=19916.3 (θ=89.3°)
13
d=22359.3 (θ=86.6°)
d=22636.7 (θ=86.7°)
d=21989.0 (θ=88.5°)
d=22716.7 (θ=87.2°)
d=19724.5 (θ=89.9°)
14

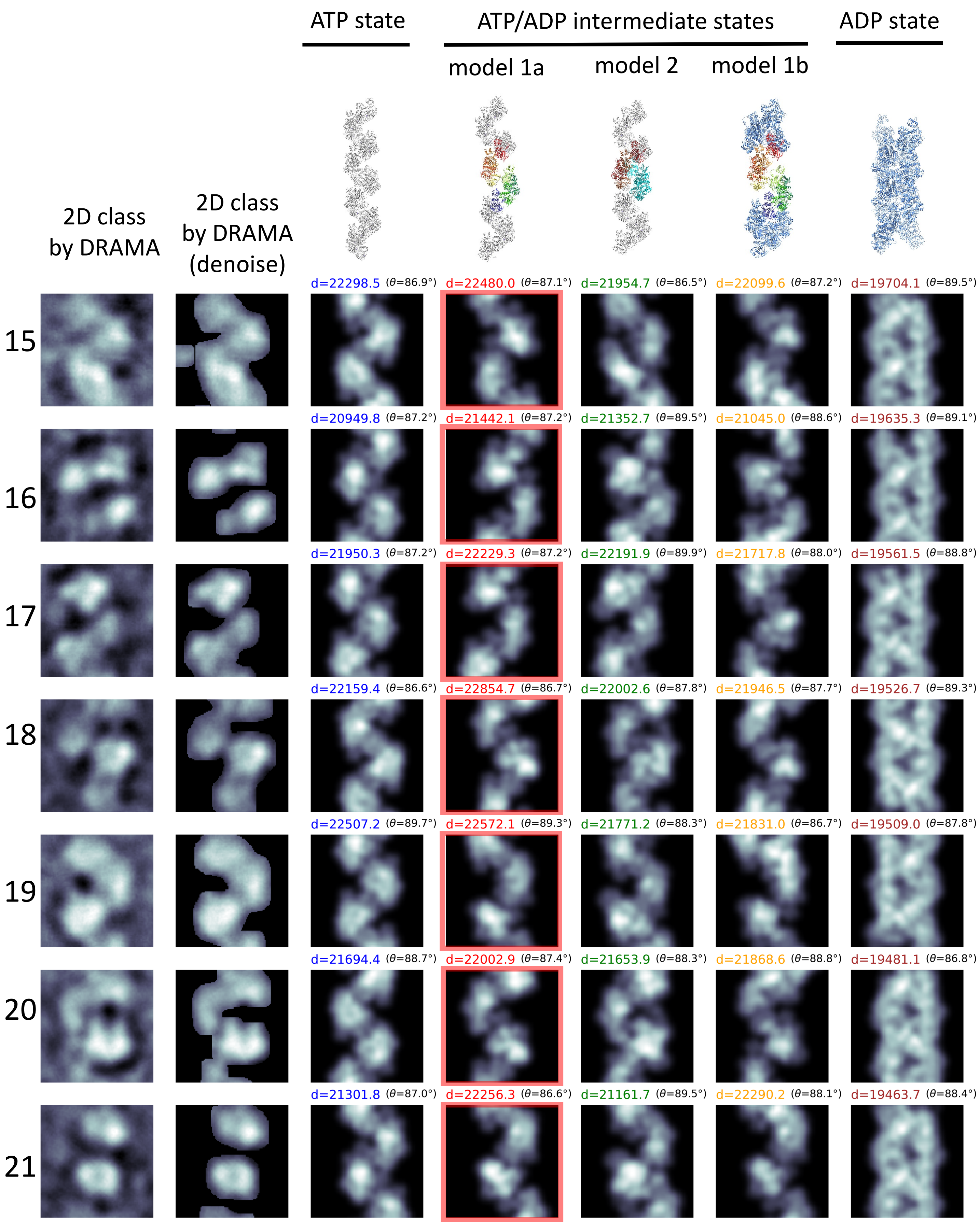

ATP state
ATP/ADP intermediate states
ADP state
model 1a
model 2
model 1b
2D class by DRAMA
2D class by DRAMA (denoise)
15
d=22298.5 (θ=86.9°)
d=22480.0 (θ=87.1°)
d=21954.7 (θ=86.5°)
d=22099.6 (θ=87.2°)
d=19704.1 (θ=89.5°)
16
d=20949.8 (θ=87.2°)
d=21442.1 (θ=87.2°)
d=21352.7 (θ=89.5°)
d=21045.0 (θ=88.6°)
d=19635.3 (θ=89.1°)
17
d=21950.3 (θ=87.2°)
d=22229.3 (θ=87.2°)
d=22191.9 (θ=89.9°)
d=21717.8 (θ=88.0°)
d=19561.5 (θ=88.8°)
18
d=22159.4 (θ=86.6°)
d=22854.7 (θ=86.7°)
d=22002.6 (θ=87.8°)
d=21946.5 (θ=87.7°)
d=19526.7 (θ=89.3°)
19
d=22507.2 (θ=89.7°)
d=22572.1 (θ=89.3°)
d=21771.2 (θ=88.3°)
d=21831.0 (θ=86.7°)
d=19509.0 (θ=87.8°)
20
d=21694.4 (θ=88.7°)
d=22002.9 (θ=87.4°)
d=21653.9 (θ=88.3°)
d=21868.6 (θ=88.8°)
d=19481.1 (θ=86.8°)
21
d=21301.8 (θ=87.0°)
d=22256.3 (θ=86.6°)
d=21161.7 (θ=89.5°)
d=22290.2 (θ=88.1°)
d=19463.7 (θ=88.4°)

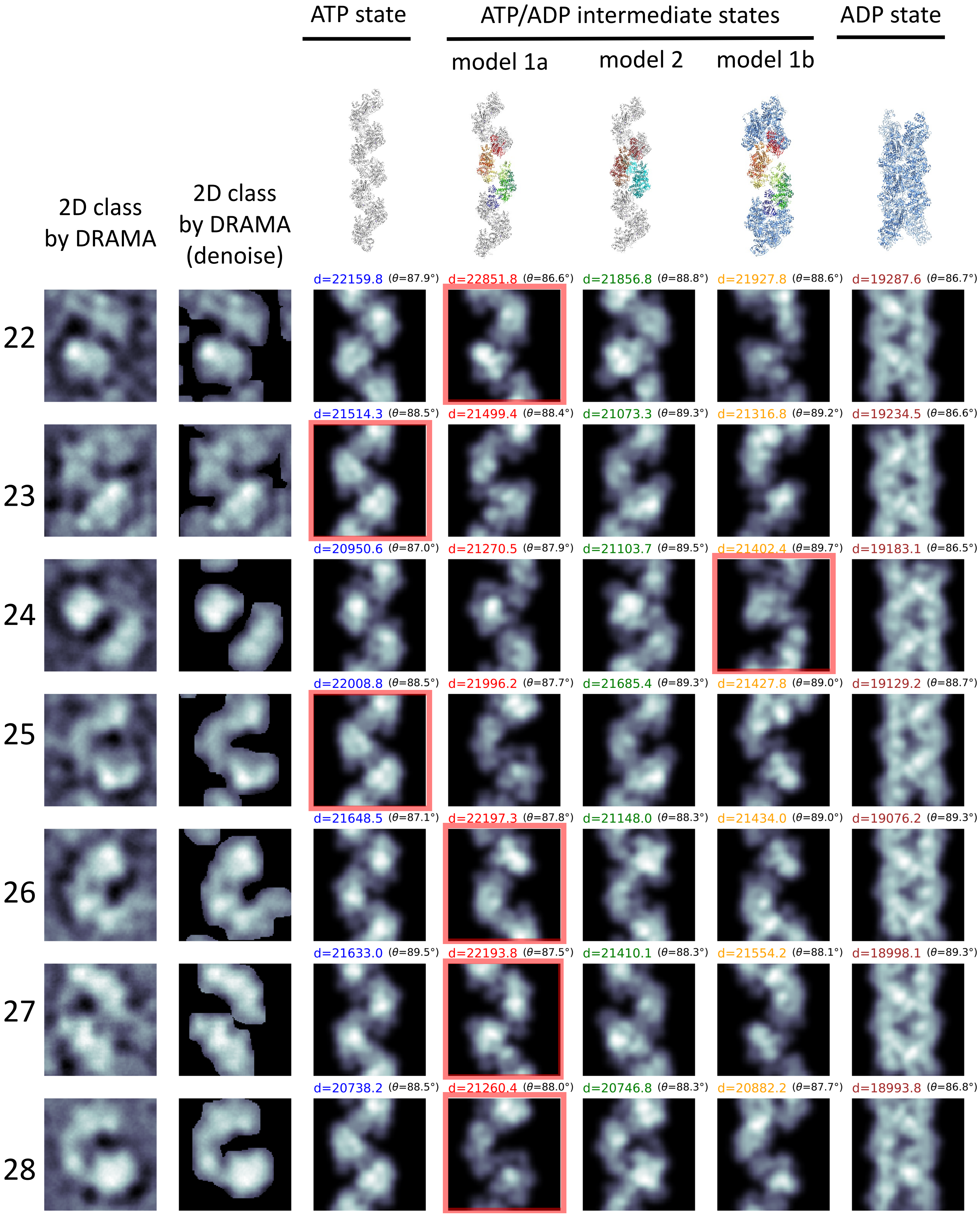
ATP state
ATP/ADP intermediate states
ADP state
model 1a
model 2
model 1b
2D class by DRAMA
2D class by DRAMA (denoise)
22
d=22159.8 (θ=87.9°)
d=22851.8 (θ=86.6°)
d=21856.8 (θ=88.8°)
d=21927.8 (θ=88.6°)
d=19287.6 (θ=86.7°)
23
d=21514.3 (θ=88.5°)
d=21499.4 (θ=88.4°)
d=21073.3 (θ=89.3°)
d=21316.8 (θ=89.2°)
d=19234.5 (θ=86.6°)
24
d=20950.6 (θ=87.0°)
d=21270.5 (θ=87.9°)
d=21103.7 (θ=89.5°)
d=21402.4 (θ=89.7°)
d=19183.1 (θ=86.5°)
25
d=22008.8 (θ=88.5°)
d=21996.2 (θ=87.7°)
d=21685.4 (θ=89.3°)
d=21427.8 (θ=89.0°)
d=19129.2 (θ=88.7°)
26
d=21648.5 (θ=87.1°)
d=22197.3 (θ=87.8°)
d=21148.0 (θ=88.3°)
d=21434.0 (θ=89.0°)
d=19076.2 (θ=89.3°)
27
d=21633.0 (θ=89.5°)
d=22193.8 (θ=87.5°)
d=21410.1 (θ=88.3°)
d=21554.2 (θ=88.1°)
d=18998.1 (θ=89.3°)
28
d=20738.2 (θ=88.5°)
d=21260.4 (θ=88.0°)
d=20746.8 (θ=88.3°)
d=20882.2 (θ=87.7°)
d=18993.8 (θ=86.8°)

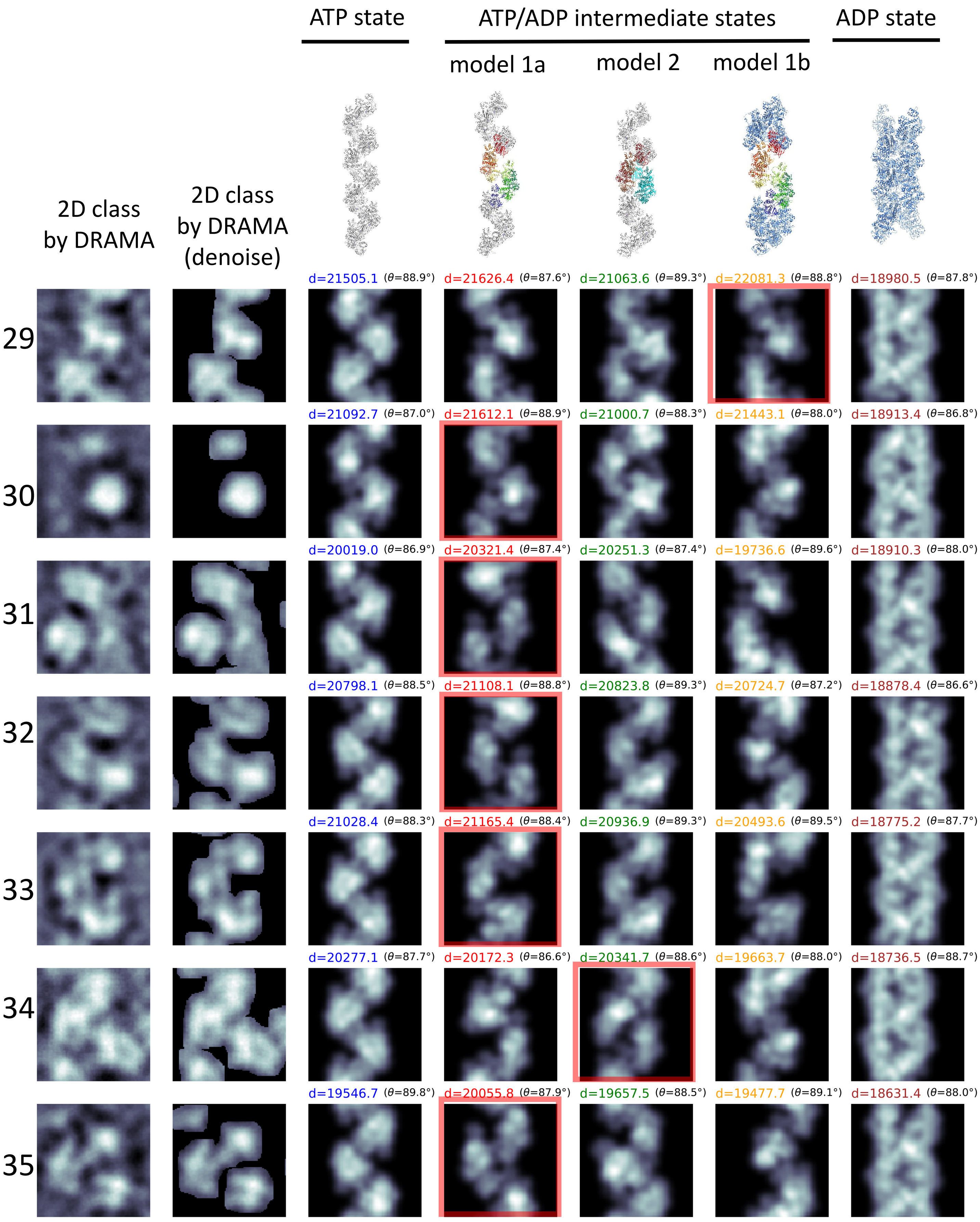
ATP state
ATP/ADP intermediate states
ADP state
model 1a
model 2
model 1b
2D class by DRAMA
2D class by DRAMA (denoise)
29
d=21505.1 (θ=88.9°)
d=21626.4 (θ=87.6°)
d=21063.6 (θ=89.3°)
d=22081.3 (θ=88.8°)
d=18980.5 (θ=87.8°)
30
d=21092.7 (θ=87.0°)
d=21612.1 (θ=88.9°)
d=21000.7 (θ=88.3°)
d=21443.1 (θ=88.0°)
d=18913.4 (θ=86.8°)
31
d=20019.0 (θ=86.9°)
d=20321.4 (θ=87.4°)
d=20251.3 (θ=87.4°)
d=19736.6 (θ=89.6°)
d=18910.3 (θ=88.0°)
32
d=20798.1 (θ=88.5°)
d=21108.1 (θ=88.8°)
d=20823.8 (θ=89.3°)
d=20724.7 (θ=87.2°)
d=18878.4 (θ=86.6°)
33
d=21028.4 (θ=88.3°)
d=21165.4 (θ=88.4°)
d=20936.9 (θ=89.3°)
d=20493.6 (θ=89.5°)
d=18775.2 (θ=87.7°)
34
d=20277.1 (θ=87.7°)
d=20172.3 (θ=86.6°)
d=20341.7 (θ=88.6°)
d=19663.7 (θ=88.0°)
d=18736.5 (θ=88.7°)
35
d=19546.7 (θ=89.8°)
d=20055.8 (θ=87.9°)
d=19657.5 (θ=88.5°)
d=19477.7 (θ=89.1°)
d=18631.4 (θ=88.0°)

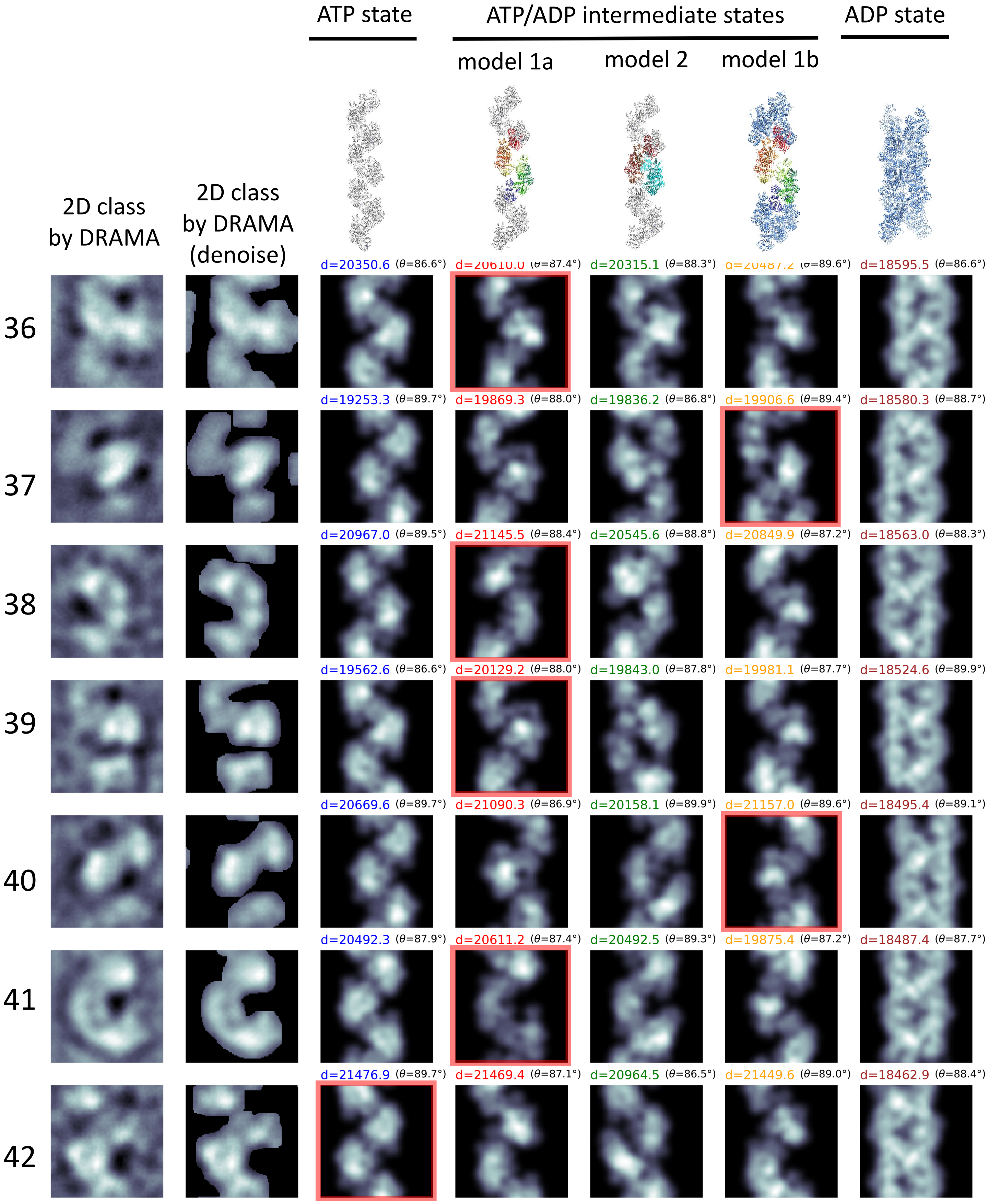
ATP state
ATP/ADP intermediate states
ADP state
model 1a
model 2
model 1b
2D class by DRAMA
2D class by DRAMA (denoise)
36
37
38
39
40
41
42
d=20350.6 (θ=86.6°)
(θ=87.4°)
d=20315.1 (θ=88.3°)
(θ=89.6°)
d=18595.5 (θ=86.6°)
d=19253.3 (θ=89.7°)
d=19869.3 (θ=88.0°)
d=19836.2 (θ=86.8°)
d=19906.6 (θ=89.4°)
d=18580.3 (θ=88.7°)
d=20967.0 (θ=89.5°)
d=21145.5 (θ=88.4°)
d=20545.6 (θ=88.8°)
d=20849.9 (θ=87.2°)
d=18563.0 (θ=88.3°)
d=19562.6 (θ=86.6°)
d=20129.2 (θ=88.0°)
d=19843.0 (θ=87.8°)
d=19981.1 (θ=87.7°)
d=18524.6 (θ=89.9°)
d=20669.6 (θ=89.7°)
d=21090.3 (θ=86.9°)
d=20158.1 (θ=89.9°)
d=21157.0 (θ=89.6°)
d=18495.4 (θ=89.1°)
d=20492.3 (θ=87.9°)
d=20611.2 (θ=87.4°)
d=20492.5 (θ=89.3°)
d=19875.4 (θ=87.2°)
d=18487.4 (θ=87.7°)
d=21476.9 (θ=89.7°)
d=21469.4 (θ=87.1°)
d=20964.5 (θ=86.5°)
d=21449.6 (θ=89.0°)
d=18462.9 (θ=88.4°)

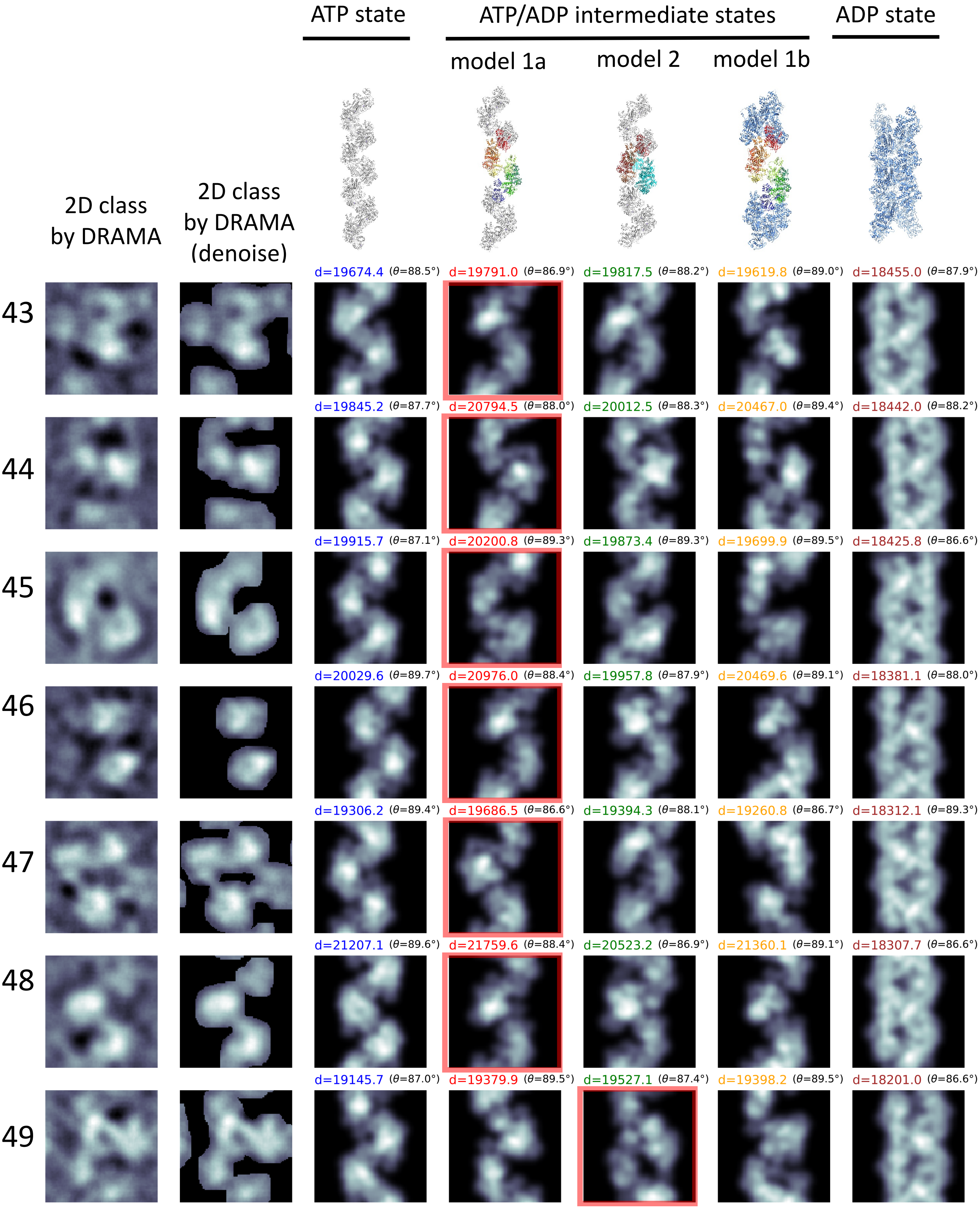
ATP state
ATP/ADP intermediate states
ADP state
model 1a
model 2
model 1b
2D class by DRAMA
2D class by DRAMA (denoise)
43
d=19674.4 (θ=88.5°)
d=19791.0 (θ=86.9°)
d=19817.5 (θ=88.2°)
d=19619.8 (θ=89.0°)
d=18455.0 (θ=87.9°)
44
d=19845.2 (θ=87.7°)
d=20794.5 (θ=88.0°)
d=20012.5 (θ=88.3°)
d=20467.0 (θ=89.4°)
d=18442.0 (θ=88.2°)
45
d=19915.7 (θ=87.1°)
d=20200.8 (θ=89.3°)
d=19873.4 (θ=89.3°)
d=19699.9 (θ=89.5°)
d=18425.8 (θ=86.6°)
46
d=20029.6 (θ=89.7°)
d=20976.0 (θ=88.4°)
d=19957.8 (θ=87.9°)
d=20469.6 (θ=89.1°)
d=18381.1 (θ=88.0°)
47
d=19306.2 (θ=89.4°)
d=19686.5 (θ=86.6°)
d=19394.3 (θ=88.1°)
d=19260.8 (θ=86.7°)
d=18312.1 (θ=89.3°)
48
d=21207.1 (θ=89.6°)
d=21759.6 (θ=88.4°)
d=20523.2 (θ=86.9°)
d=21360.1 (θ=89.1°)
d=18307.7 (θ=86.6°)
49
d=19145.7 (θ=87.0°)
d=19379.9 (θ=89.5°)
d=19527.1 (θ=87.4°)
d=19398.2 (θ=89.5°)
d=18201.0 (θ=86.6°)

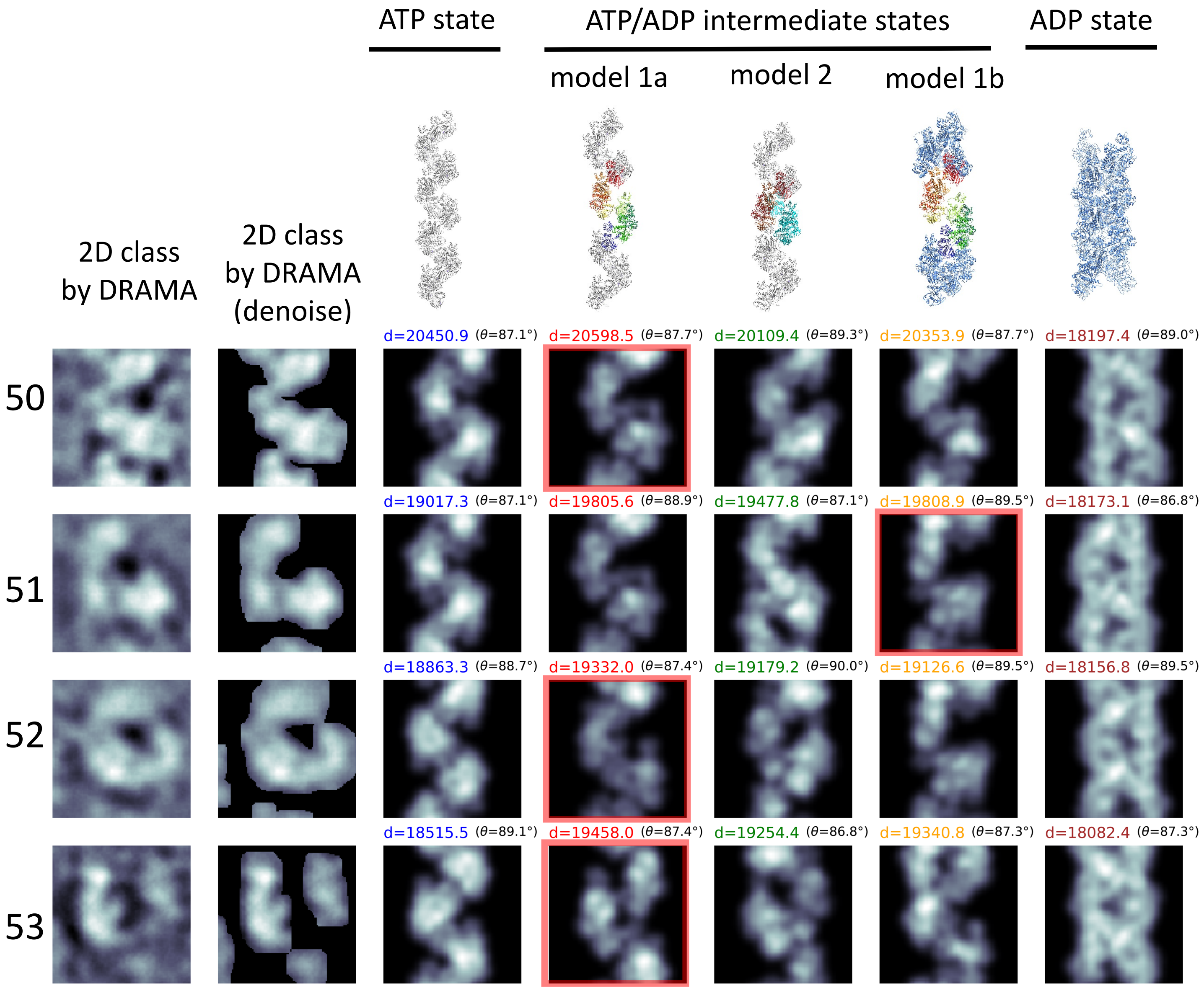

ATP state
ATP/ADP intermediate states
ADP state
model 1a
model 2
model 1b
2D class by DRAMA
2D class by DRAMA (denoise)
50
d=20450.9 (θ=87.1°)
d=20598.5 (θ=87.7°)
d=20109.4 (θ=89.3°)
d=20353.9 (θ=87.7°)
d=18197.4 (θ=89.0°)
51
d=19017.3 (θ=87.1°)
d=19805.6 (θ=88.9°)
d=19477.8 (θ=87.1°)
d=19808.9 (θ=89.5°)
d=18173.1 (θ=86.8°)
52
d=18863.3 (θ=88.7°)
d=19332.0 (θ=87.4°)
d=19179.2 (θ=90.0°)
d=19126.6 (θ=89.5°)
d=18156.8 (θ=89.5°)
53
d=18515.5 (θ=89.1°)
d=19458.0 (θ=87.4°)
d=19254.4 (θ=86.8°)
d=19340.8 (θ=87.3°)
d=18082.4 (θ=87.3°)
