# Supplementary material for: A RAD51–ADP double filament structure unveils the mechanism of filament dynamics in homologous recombination
Source: Nat Commun. 2023 Aug 17;14:4993. doi: 10.1038/s41467-023-40672-5 (PMC10435448; doi:10.1038/s41467-023-40672-5)
Supplement: Supplementary file 3 — Description of Additional Supplementary Files [file 41467_2023_40672_MOESM3_ESM.pdf]

Description of Additional Supplementary Files

File Name: Supplementary Movie 1
Description: Inter-protomer movement between hRAD51–ATP and hRAD51–ADP

File Name: Supplementary Movie 2
Description: Conformational changes around the nucleotide-binding area between hRAD51–ATP and hRAD51–ADP

File Name: Supplementary Movie 3
Description: Movie presentation of the collapsing mechanism allowing stepwise movements while the ADP-bound filament remains attached to the adjacent one

File Name: Supplementary Movie 4
Description: Movie presentation of collapsing mechanism in the reversible direction

File Name: Supplementary Movie 5
Description: Movie presentation of steric hindrance occurring in two three-promoter movements
