# Supplementary material for: A RAD51–ADP double filament structure unveils the mechanism of filament dynamics in homologous recombination
Source: Nat Commun. 2023 Aug 17;14:4993. doi: 10.1038/s41467-023-40672-5 (PMC10435448; doi:10.1038/s41467-023-40672-5)
Supplement: Supplementary file 9 — Reporting Summary [file 41467_2023_40672_MOESM9_ESM.pdf]

nature portfolio

Corresponding author(s): Meng-Chiao Ho
Last updated by author(s): Jun 9, 2023

Reporting Summary

Nature Portfolio wishes to improve the reproducibility of the work that we publish. This form provides structure for consistency and transparency in reporting. For further information on Nature Portfolio policies, see our Editorial Policies and the Editorial Policy Checklist.

Statistics

For all statistical analyses, confirm that the following items are present in the figure legend, table legend, main text, or Methods section.

n/a	Confirmed	
☐	☒	The exact sample size (*n*) for each experimental group/condition, given as a discrete number and unit of measurement
☐	☒	A statement on whether measurements were taken from distinct samples or whether the same sample was measured repeatedly
☐	☒	The statistical test(s) used AND whether they are one- or two-sided<br>*Only common tests should be described solely by name; describe more complex techniques in the Methods section.*
☐	☒	A description of all covariates tested
☐	☒	A description of any assumptions or corrections, such as tests of normality and adjustment for multiple comparisons
☐	☒	A full description of the statistical parameters including central tendency (e.g. means) or other basic estimates (e.g. regression coefficient) AND variation (e.g. standard deviation) or associated estimates of uncertainty (e.g. confidence intervals)
☒	☐	For null hypothesis testing, the test statistic (e.g. *F*, *t*, *r*) with confidence intervals, effect sizes, degrees of freedom and *P* value noted<br>*Give P values as exact values whenever suitable.*
☒	☐	For Bayesian analysis, information on the choice of priors and Markov chain Monte Carlo settings
☒	☐	For hierarchical and complex designs, identification of the appropriate level for tests and full reporting of outcomes
☒	☐	Estimates of effect sizes (e.g. Cohen's *d*, Pearson's *r*), indicating how they were calculated

Our web collection on statistics for biologists contains articles on many of the points above.

Software and code

Policy information about availability of computer code

Data collection	Phosphorimaging analysis (Bio-Rad Personal Molecular Imager): EMSA<br>Cryo-EM data were acquired on a Titan Krios G3 (Thermo Fisher) microscope operated at 300 keV, equipped with a GIF Quantum K3 dector system (Gatan) or a Talos Arctica (Thermo Fisher) microscope operated at 200 KeV, equipped with a Falcon III detector. Automated data acquisition was carried out using EPU software (Thermo Fisher).
Data analysis	Quantitative analyses for phosphorimages were performed using ImageQuant TL.<br>CryoEM data processing was motion-corrected and dose-weighted using RELION3.0's own implementation. Further data processing and helical reconstruction was performed by cryoSPARC. CryoEM structure refinement was done with PHENIX. The refined atomic models were further validated and manually inspected using PHENIX and COOT.<br>RE2DC, a robust and efficient 2D classification algorithm, was used to allow effective 2D class averaging of the intermediate state from a limited amount of particle images per class.

For manuscripts utilizing custom algorithms or software that are central to the research but not yet described in published literature, software must be made available to editors and reviewers. We strongly encourage code deposition in a community repository (e.g. GitHub). See the Nature Portfolio guidelines for submitting code & software for further information.

Data

Policy information about availability of data

All manuscripts must include a data availability statement. This statement should provide the following information, where applicable:

- Accession codes, unique identifiers, or web links for publicly available datasets
- A description of any restrictions on data availability
- For clinical datasets or third party data, please ensure that the statement adheres to our policy

The structure coordinates and cryo-EM maps have been deposited in the Protein Data Bank and the Electron Microscopy Data Bank under accession codes 8GYK and EMD-34372 (focused-refined map), and 36040 (composite map). All additional coordinate and low-resolution cryo-EM maps related to our proposed collapsing mechanism have been deposited in https://doi.org/10.6084/m9.figshare.20920903. The Source Data file consists of raw data and uncropped gel images for Figure 3. Other additional data related to this paper are available upon request.

Human research participants

Policy information about studies involving human research participants and Sex and Gender in Research.

Reporting on sex and gender	No human research is reported in this manuscript.
Population characteristics	See above.
Recruitment	See above.
Ethics oversight	See above.

Note that full information on the approval of the study protocol must also be provided in the manuscript.

Field-specific reporting

Please select the one below that is the best fit for your research. If you are not sure, read the appropriate sections before making your selection.

☒ Life sciences ☐ Behavioural & social sciences ☐ Ecological, evolutionary & environmental sciences

For a reference copy of the document with all sections, see nature.com/documents/nr-reporting-summary-flat.pdf

Life sciences study design

All studies must disclose on these points even when the disclosure is negative.

Sample size	Biochemical data shown were from at least three independent experiments. The sample size was sufficient to represent reproducibility and credibility in the biochemical assay. Numbers of particles in the cryoEM samples are described in the methods.
Data exclusions	CryoEM data processing, including which particles are included or excluded from the final map, is described in the methods. Flow charts are shown in the supplementary figure 2, 3 and 12.
Replication	EMSA experiments were reliably reproduced. The data were analyzed from three independent experiments.
Randomization	CryoEM particles were randomly assigned to two half-sets, following the "gold standard" FSC protocol. Randomization was not relevant to biochemical assay and was not performed for biochemical experiments.
Blinding	Blinding was not essential to the biochemical assay and cryoEM.

Reporting for specific materials, systems and methods

We require information from authors about some types of materials, experimental systems and methods used in many studies. Here, indicate whether each material, system or method listed is relevant to your study. If you are not sure if a list item applies to your research, read the appropriate section before selecting a response.

Materials & experimental systems

n/a	Involved in the study
☒	☐ Antibodies
☒	☐ Eukaryotic cell lines
☒	☐ Palaeontology and archaeology
☒	☐ Animals and other organisms
☒	☐ Clinical data
☒	☐ Dual use research of concern

Methods

n/a	Involved in the study
☒	☐ ChIP-seq
☒	☐ Flow cytometry
☒	☐ MRI-based neuroimaging
